# Enhancing socio-communicative functions in an MCI patient with intra-nasal insulin: a case report

**DOI:** 10.3389/fpsyt.2024.1326702

**Published:** 2024-06-28

**Authors:** Sara Schatz, Grace Rose Gutiérrez

**Affiliations:** ^1^ International Studies, The Ohio State University, Columbus, OH, United States; ^2^ Department of Evolution, Ecology, and Organismal Biology, The Ohio State University, Columbus, OH, United States

**Keywords:** intranasal insulin, socio-communicative abilities, MCI (mild cognitive impairment), Alzheimer’s dementia, cognitive improvement

## Abstract

This report examines extended intra-nasal insulin treatment [INI] for an Insulin Resistant early Mild Cognitive Impairment [MCI] patient. Patient [EJ] also had medial temporal lobe [MTL] damage, poor short-term memory, significant irritability, and social and linguistic withdrawal at treatment start. Compared to baseline, nine months INI treatment increased grey matter volume, lowered beta-amyloid levels, and improved MCI and FAS scores. Patient also increased pragmatic capacities in social conversation and procedural memory. These findings align with results from prior clinical trials on INI and suggest that treatment can slow neurodegenerative disease progression in early MCI patients.

## 1 Report structure

Section II reviews intra-nasal insulin treatment [INI] mediated improvements in pragmatic competence and lessened social isolation from other speakers. Section III discusses patient [EJ] history and trajectory which involved hyperglycemia, insulin resistance (IR), short-term memory deficits and grey matter volume (GMV) reduction years before initial mild cognitive impairment (MCI) diagnosis. Section IV details EJ’s increased pragmatic capacities and cognitive load under INI. Section V discusses how INI improves pragmatic competence as related to executive functioning.

## 2 INI improves pragmatic competence in MCI and AD

### 2.1 INI therapy & socio-communicative functioning in MCI & AD-cognitive & language decline

Clinical studies support that short-term INI improves cognitive capacities and quality of life for patients with Diabetes Mellitus (Type II, T2DM), MCI, and Alzheimer’s Disease (AD). INI improved episodic memory, verbal working memory and short-term serial memory after 3–4 months (1, 2). After twelve months treatment, INI increased memory consolidation and cognition (attention, reasoning, orientation, praxis, language) in patients with MCI and mild Alzheimer’s Disease (3, Device I)^1^.

Studies of MCI, early and moderate AD patients found INI treatment increased pragmatic competence and reduced social isolation from other speakers (4–6). Models of cognitive impairment and pragmatics conceptualize pragmatic competence in relation to the expressive modality. Expressive modality is the ability to express meaningful utterances (illocutionary force) and successfully achieve appropriate effects of expressions (perlocutionary effect) upon listeners (7). Pragmatic abilities also refer to the decoding of the content of utterances (8). Executive functioning includes internally directed attention, introspection, and greater capacity for externally directed attention (9). Communicative participation is ‘involvement in a life situation where knowledge, information, ideas or feelings are exchanged. It may take the form of speaking, listening, reading, writing, or nonverbal means of communication … and must involve a communicative exchange’ (10:309).

When these domains become linguistically impaired, in language-based everyday tasks it can be expressed in the progressive loss of ability to communicate, beginning with early AD language deficits (word substitutions, aborted phrases), then progresses to comprehension deficits, paraphrasic errors, and semantic jargon in mid-to-late stage Alzheimer’s (11:63). Semantic and lexical speech errors multiply, along with a greater number of personal pronouns, incomplete sentences and empty pauses (12). Speech becomes formulaic and the patient might even produce appropriate utterances even when doubt arises as to whether they are really meant (13).

In the study of day-to-day communication in MCI and AD, pragmatic ability includes the domain of executive functioning, and is inextricably associated with cognitive, linguistic and sensorimotor elements in the intrapersonal areas which it controls (4–6)^2^. INI treated patients increased illocutionary and perlocutionary abilities such as humor, irony and sarcasm (6, 14). INI even improved pragmatic capacity in a late-stage AD patient with a severe cognitive communication disorder (akinetic, abulic type) at baseline (14).

Untreated, declines of linguistic aspects of AD terminate in complete mutism and high caregiver stress (11), a cycle of social isolation from other speakers. In this cycle, the patient experiences depression, confidence loss, and altered power relationships. As the disease progresses, the patient becomes isolated from other speakers. Caregivers often speak negatively about the patient to others even when the patient is also present, as if they were not there. Heightened caregiver stress is chronic, widespread, and accompanies progressive losses of communication ability in AD (15). Aggression in communication can emerge from the unresolved non-alignment of communicative purpose and/or achievement, culminating in outward expression of physical, linguistic or emotional force against someone else (16). Pragmatic dissonance may emerge or worsen when an MCI or AD patient’s behavior no longer fulfills the (previous) expectations of a caregiver, spouse, child or family member.

In MCI and AD patients at all stages of disease progression, INI improves attention, emotional self-awareness, and empathy. These improvements facilitate engagement in pleasureful social interaction and reduce family and caregiver stress (4:331–337; 5:375–378; 381–383; 6:7–11). For example, a late-stage AD patient [KG] had severe akinetic mutism (abulic type) pre-treatment and provided monosyllabic, affect-less, incorrect answers to questions expressed in full sentences (“Did you eat your breakfast?” “No”, “Did you watch TV” “No” even though she did). In contrast, after INI, KG made socially appropriate comments and expressed appropriate affect (“I love you”, “You work hard”, “*I like this*”) (14). KG began staying awake during home movies, communicating self-recognition to caregivers (“I remember that”), whereas she had ceased to do so before INI. Her expressions elicited immediate positive caregiver responses (“She is speaking!”, “She has really improved”, “She is paying attention”, laugher) as the patient achieved appropriate perlocutionary effects.

### 2.2 HbA1c, cognitive decline (IR, pre-diabetes, T2DM) & INI therapy

IR is a significant risk factor for neurodegeneration and remains a challenge. Symptoms of IR often begin 20–30 years before MCI is diagnosed (17). IR underdiagnosis occurs in medical practice due to lack of solid assays for IR and because patients and doctors are often unaware that HbA1c may be a useful proxy to assess IR-related negative effects on cognition (18). In some cases, patients can have insulin levels 300 times above normal but glucose levels that do not exceed the threshold for diabetes. As Craft (18) notes, such patients “are told by their practitioners that they are fine. So, the awareness that one should have their IR assessed as well as their glucose is a very important message”.

HbA1c scores are correlated with memory formation and retention (19). Pre-diabetes and Type 2 diabetes mellitus (T2DM) accelerate memory loss, brain aging and increase risk for dementia via inflammation, impaired insulin-mediated brain signaling and oxidative stress (2, 20, 21). Insulin is a key neuroprotector in the brain, modulating energy metabolism, neurovascular coupling, promoting regeneration and preventing damage induced by ischemia, β-amyloid toxicity, oxidative stress, and apoptosis (22). Higher baseline BMI is associated with higher baseline insulin resistance (23:902) and obesity is a triggering factor for T2DM associated with insulin resistance (24).

As a therapy for IR and T2DM, INI stimulates dopaminergic and hypothalamic pathways after it enters the brain, bypassing the blood–brain barrier, along olfactory, trigeminal pathways and perivascular channels, binding to receptors in multiple cortical regions, hypothalamus, cerebellum, insula, hippocampus and substantia nigra (25). Even a single 40 IU dose of INI in T2DM patients demonstrated increased resting-state connectivity between the hippocampal regions and the medial frontal cortex as compared with placebo (26). This connectivity regulates and improves memory and complex cognitive behaviors including attention span, verbal and visuospatial memory in older T2DM and healthy adults (26, 27).

## 3 Patient history & the long road to diagnosis

Section III outlines EJ’s pre-INI trajectory of early brain insulin resistance to MCI diagnosis.

### 3.1 High glucose levels for 14 years

EJ is a retired, 80-year-old mechanical engineer with a doctorate. He is married for 52 years with two adult daughters. He and his wife live in their own home.

**Figure 1:**
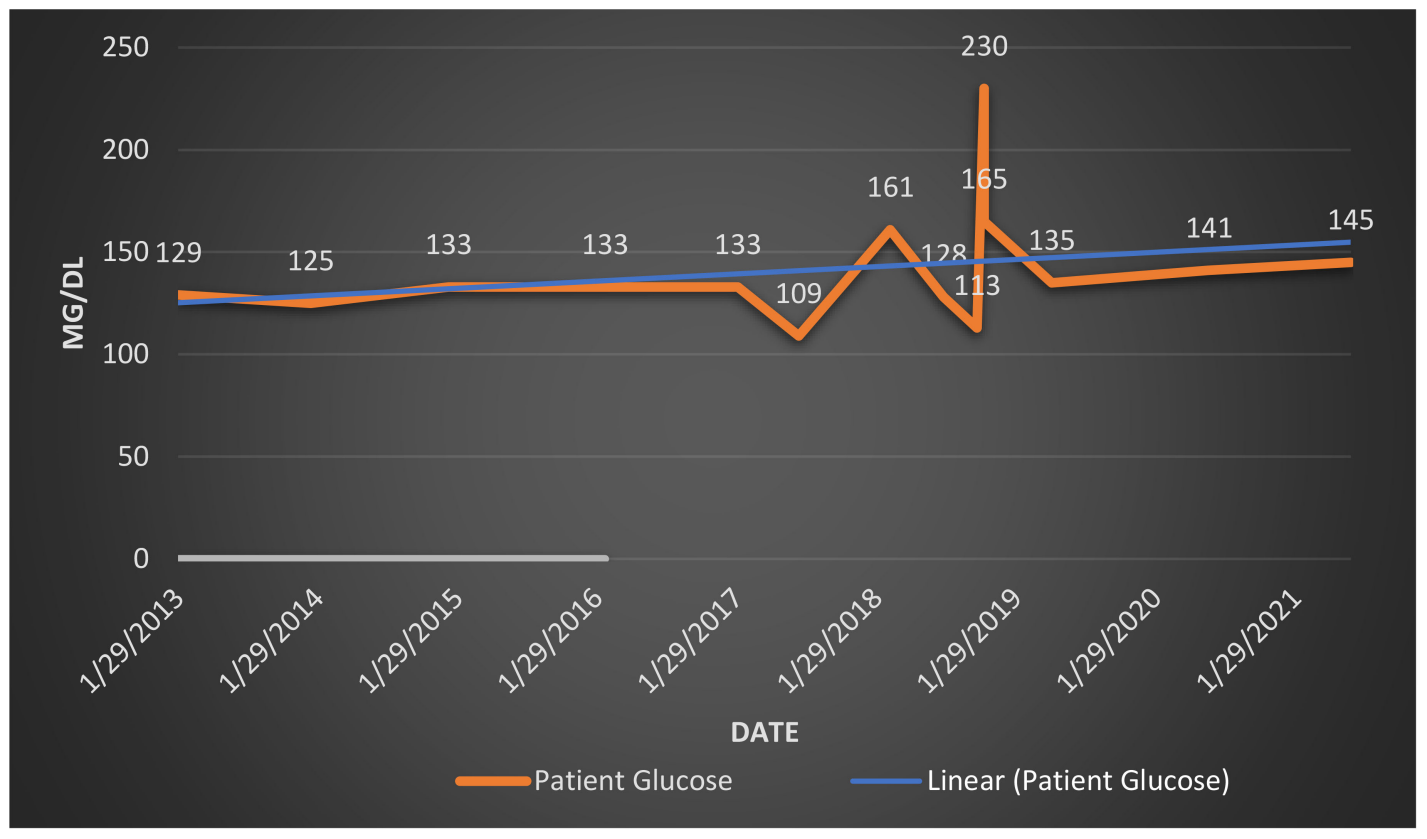
Patient glucose levels, age 70–78. Graph of patient glucose (mg/dl-milligrams per deciliter).

EJ’s existing medical records show he had abnormal blood sugar fourteen years before he was clinically diagnosed with MCI on October 12, 2020. From 2006 to 2010, EJ’s fasting glucose levels were in a lesser elevated range (101–115 mg/dL, 28) with a median score of 116mg/dL^5^ (ages 64–69, patient records). By 2013, EJ was hyperglycemic; his glucose levels were frequently over the normal range of 100–125 mg/dL (28, **Figure 1**), and fluctuated at the 125–230 range with a median score of 142 mg/dL (ages 70–78). Yet, despite these abnormalities, his 2016 doctor’s notes reported: “Overall health looks very good^3^.”.

### 3.2 Family awareness of short-term related memory loss

EJ’s wife first described EJ’s short-term memory loss as follows: “The first time I saw it was about a year ago. I was at home and EJ had a letter to take out to mail. He went out to do errands and came back several hours later with the letter still in his pocket. It was sticking out and he had forgotten to mail it (Personal Interview, 5/24/21)”.

EJ’s wife had difficulty comprehending his cognitive decline. “He would get upset even if something was a minor issue. Technology became even more of an issue for him. He forgets things that he knew before like how to work Amazon, shared pill prescription, addresses”. She felt it was “almost like he did not want to share something and then he would be confused, like he was hiding or in denial” (Personal Interview, 5/24/21). Task execution was also problematic as he would forget the second step in two-step processes. “If I said, ‘put these bags in the car, then go to the garage and grab the ladder’, he would do the bags but then forget the second step and come back and ask for guidance”.

Procedural memory refers to repetitive, cognitive task-based learning. EJ’s difficulties with procedural memory were highly stressful for his family. EJ’s daughter recognized declines in her father’s task execution abilities; he was unable to complete her two-phased monthly medical bill payment. He deteriorated to the point that she had to re-explain it many times and now pays the bill herself. His confusion and forgetfulness were stressful for her (“It is frustrating, like beating a dead horse”). EJ’s wife reported that he had become increasingly verbally combative with her. Feeling as though he frequently blamed her for daily inconveniences, she had begun regularly avoiding him.

### 3.3 Pre-diabetes & MCI diagnosis

**Figure 2:**
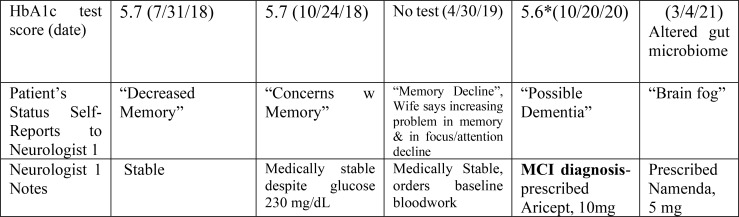
Patient HbA1c score and patient status history.

EJ received minimal HbA1c testing prior to 2021. Subsequent HbA1c scores revealed his pre-diabetic status (**Figure 2**, patient medical records)^4^ ^,^ ^5^.

Interestingly, EJ’s fluctuating pre-diabetic status correlated with his self-reporting of increasing signs of memory loss before his formal MCI diagnosis (10/12/20). As **Figure 2** shows, EJ persisted for two years and 5 months in expressing to neurologist (#1) his “decreased memory” (5/31/18), “concerns and changes with [his] memory” (1/29/19), “memory decline” which his wife also linked to his reduced focus and attentional abilities (4/30/19), and finally “possible dementia” (10/12/20) before his formal MCI diagnosis. Patient began 10mg daily Aricept on 10/20/20. Five months later (3/4/21), EJ experienced bowel changes and increased bloating and gas. These changes are consistent with an altered gut microbiome found to play a key role in the pathogenesis of neurological disorders, including the transition from MCI to AD (30). EJ also complained of episodes of ‘brain fog’ by which he meant more constantly not remembering the location of the doctor’s office or finding paperwork that was in his car and losing his phone and glasses. Patient began 5mg daily Namenda on 3/4/21.

### 3.4 Baseline MRI & MCI results

**Figure 3:**
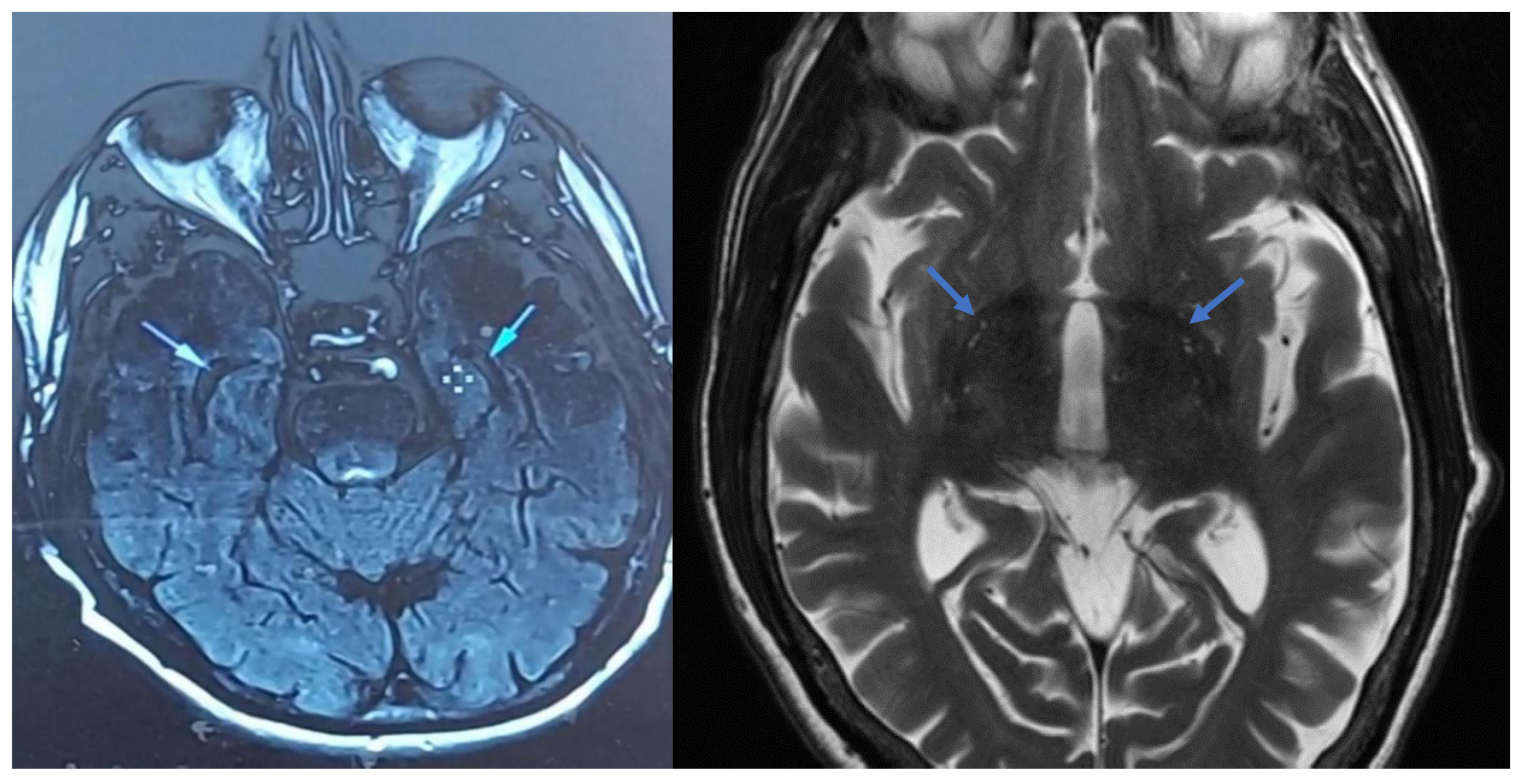
Temporal lobe ventricles change in grey matter volume between baseline and treatment month 9 (cross-sectional view). Left image: Treatment baseline (6/21/21). Right image: After 9 months of treatment (3/11/22).

In mid-2021, EJ and his family were referred to a neurological clinic which specializes in INI treatment for memory disorders due to deteriorating memory and heightened family stress. The new treating neurologist (#2) diagnosed EJ with IR/T2DM due to his HbA1c test (5.9, Borderline High, 7/22/21),^5^ long-standing hyperglycemia (glucose levels, 145 mg/dL (7/22/21), Baseline MCI Screen (**Figure 3A**, 6/15/21, Below Normal, Section 4.1.10) and Baseline MRI (6/21/21, 1 single small stroke [lacunar infarct]).

EJ’s MRI (6/21/21) also showed abnormal grey matter volume [GMV] loss at the tips of the temporal lobes (**Figure 3A**, Sec. 4.1.10.). This loss is shown by black spaces on lateral ventricles. EJ’s MRI results are consistent with findings that higher IR is significantly associated with less GMV in the medial temporal lobe [MTL]. Willette et al. (31) reported positive associations between IR and shifts from MTL-hyper to hypometabolism.

### 3.5 Hormonal panel & other screening

Patient was also diagnosed with severe testicular hypofunction (6/22/21; 2.3 Free Testosterone (T), LH High at 15.9). EJ’s testicular condition had been previously undetected and low T has been associated with a 50% higher risk of developing AD (32). Patient began topical testosterone gel (1.62%; 6/21) which incrementally increased his Free Testosterone Levels (2.3c to 2.5 6/22/21–8/9/21) to normal (8.0, 7/29/22). Patient was also negative for APOE and sleep apnea (3/5/22).

## 4 INI treatment and associated patient outcomes

### 4.1 Methods

Section IV discusses EJ’s increased pragmatic and cognitive load and communicative participation under targeted INI. Data in this section originated from written and audio recordings of communicative interactions between EJ, his family, friends, and the researcher in the home of EJ’s daughter. Such interactions include EJ’s verbalized self-perceptions, self-reflections, discussion of daily routines, use of humor and sarcasm and cognitive task-related behaviors under INI. Data also includes interviews and observations made by the researcher on EJ’s linguistic and non-linguistic signs and their context.

Communicative interactions were interpreted using the Schiffrin (33) discourse analytic frame for the study of utterances as social interaction. Schiffrin (33) developed a framework of five components for investigating discourse: exchange structure (turn-taking), action structure (organization of speech acts such as humor, irony and sarcasm), ideational structure (the relation between propositions or ideas), information state (the organization of information and knowledge shared between conversational partners), and participation framework (how conversational partners relate to each other and to the situation with words and actions).

### 4.2 Treatment timeline & immediate treatment effects

The patient’s first treatment was administered on June 18, 2021. The treatment for the entire study duration was 40 IU of human insulin (Novolin R Novo Nordisk, Bagsværd, Denmark) delivered twice daily. MCI results are reported from baseline to 15 months after treatment initiation (September 2022). Insulin was administered intra-nasally using a ViaNase device (Kurve Technologies, Lynnwood, WA, USA). The ViaNase device used is an atomizer which transports insulin to the brain via the upper nasal cavity olfactory region. ViaNase technologies were used in multiple clinical trials to administer insulin and peptides (e.g., 1 and 3, 34).

A half-hour after this first treatment, EJ asked for a sandwich. The patient demonstrated high self-awareness^6^ as he explained in detail how he felt:

“I am feeling like I just got out of a swimming pool … In my forehead, it is a little bit of cloudiness; sluggishness-a bit heavy in the eyes. It is like I cannot get fully awake. I have to say that some of this was on my way coming over to my daughter’s house, but it seems more intense”. This implies slightly improved (self) focused attention.

EJ discussed difficulty following instructions on administering the intra-nasal insulin atomizer (a series of unfamiliar tasks): “I am following your conversations today … but I am glad that [his wife’s name] is writing down the instructions”. After the second INI administration, EJ’s wife reported that her husband had seemed “a little more helpful”, and “was able to walk the dog” that day. Nevertheless, she noted that he still became irritable when she put the car in the garage and shut the garage door without telling him.

After treatment day two, EJ’s daughter enthusiastically reported that he was able to complete, for the first time since MCI diagnoses, a two-step process. He took her ash tray with cigarette butts to the garbage can, realized the garage door was closed, then returned to the kitchen and exited out the side door (the route less commonly used) to empty the ash tray outside. When asked about this event, the patient himself said he remembered that he “threw the ash tray away” but did not remember exiting the house. EJ’s completion of this two-step process suggests increased capacity for cognitive load, attention span (vigilance) and working memory (even if not recalled by the patient).

On day three of treatment, the patient greeted the researcher, smiling, and declared: “I am feeling good, I am alert, I slept well”. His wife reported that they stayed up and watched two movies the night before, whereas usually he would fall asleep by 10pm. This behavioral change suggests increasing focus in EJ’s attention span. His wife was surprised when EJ found his misplaced glasses and remembered information that he had read the day before in Scientific American, implying greater vigilance in attention as well as greater short-term memory recall. EJ still reported a “hazy, swimming pool feeling” but said the effect was “less so”.

On treatment day four, EJ reported that he had a “good day at Walmart”. Though he still reported being “confused”, he noted that nothing “good or bad” had happened that day. The researcher observed increased communication between EJ and his family that day, implying greater pragmatic capacity and socio-communicative functioning. EJ declared: “Today, I am feeling good” while noting his efforts to argue less with his wife and “try not to get mad” (6/20/21).

EJ’s ability to articulate self-awareness is an important, if subtle, treatment-mediated gain in pragmatic capacity. EJ’s statement, “I try not to get mad”, shows an increased ability to perceive and verbally express empathy and respond to another person’s emotional state. Empathy and decreased irritability are initial INI effects found in other patients (4:332–336). These initial days of INI treatment also suggest improved focused attention and vigilance after INI intervention.

### 4.3 Months 1–2: alert, a rising cognitive load

During month one of treatment, EJ’s wife reported that he was more alert and “in a better mood” during mornings. “He complains a lot less and is a lot less grouchy”.

This couple had a long history of yearly successful international travel. In the first month of treatment, EJ’s wife took an international cruise with him, expecting his functioning to return to pre-MCI diagnosis levels. She realized after international travel was no longer feasible with EJ. She reported a great deal of pre-trip confusion for him around packing, not realizing where they were going, following instructions, and even an inability to remember his own zip code. On the cruise ship, friends traveling with them told her “They understood why [she] was giving him the treatmen.t” Still, EJ was able to socially engage at dining events despite needing his wife’s constant management of his documents, phone and personal effects in travel.

Upon returning home from the cruise, EJ’s wife noted his new difficulties remembering the location of kitchen utensils. He completed fewer daily activities, such as playing golf and reading.

### 4.4 Month 2: Patient completes complex serial tasking, greater selective attention, increased pragmatic competence (resistance, sarcasm & self-expression)

By treatment month two, EJ’s wife, to the surprise of the researcher, allowed the patient to administer his own INI for the first time (late July). EJ’s wife said she now felt “confident” in handing him responsibility for self-administering the treatment. When questioned by the researcher as to EJ’s serial tasking ability, i.e., his capacity to remember each step in executing his treatment, she responded: “I taught him repeatedly, I have all the syringes prepared with the correct dosage and he has been watching me.” Administration of INI requires 5–6 serial steps (insertion of insulin, correct positioning of atomizer to nose, accurate running of the atomizer for the correct time, proper cleaning and storage of the atomizer). According to his daughter, EJ developed a system of handwritten notes to remember this process. After placing the atomizer in the refrigerator, he indicated on the note that he had completed both treatments that day. According to his wife and daughter, EJ had developed a complex folder record system of daily treatment administration.

By month two other INI-mediated effects were noted by his daughter. She reported that her father said he was “feeling better”, especially when he administers his treatment, that treatment provides “a burst of energy” and “wakes him up”. EJ himself reported that he no longer must drink tea at night because “the treatment is helping me feel less tired and overall more awake”. EJ reported getting “good” sleep.

His daughter also said that life with her father was now “less stressful” and his ability to conduct serial tasking had increased. EJ was again able to make a password and login into his own medical records alone. He was learning and emailing her new information and calling to discuss if she had read it (7/12/2021). EJ’s daughter appreciated these interactions with her father. EJ’s greater sequential task-oriented cognitive capacities and greater self-expression had thus decreased care-giver stress by treatment month two.

The researcher administered MMSE at pre-treatment (5/18/21, score 28) and at two months treatment (8/5/21, score 29). At pre-treatment, EJ was completely unable to count backward by 7s from 100, answering uncertainly “97, 84, 91, 81, 77, 69”? In contrast, at two months, he counted perfectly backwards by 7s from 100. At pre-treatment, the only comment EJ made during the test was “my eyes are not too good either.” By month two, EJ interjected several comments during the test: “This seems ridiculously easy, but that is the test though”; “I hope I never get where I cannot do this, unless there is a trick to it.”.

On 8/5/21, the researcher asked EJ: “You had some questions, like where you were in terms of the disease stage. What would you say your questions are now?” EJ responded: “Ok, these MRI results, and I heard the doctor the other day when he described this and that and everything. You know, what I am looking for more than anything else, you know is: Do we know where I was two years ago, three years ago. Do we know where we are today? And we have some idea of the change, ok. That is, I think, quite logical. So, maybe we can never pull that data together because we didn’t really test three years ago.”.

Patient then asked questions to the researcher about what he should expect from, and how to measure, INI-mediated changes on a weekly basis as well as his future prognosis. EJ wanted more information about his health and expressed accurate inferential reasoning about the past, present and the future of his prognosis. These processes require attention, ability to processes multiple sources of information, and capacity to describe one’s personal narrative, i.e., executive-functioning. EJ had also been put in sole charge of taking care of the dog which involved administering medication daily.

Yet, EJ’s short-term memory remained limited. He noted that he had forgotten his pills when he left home to travel to his daughter’s house. Short-term memory deficits such as these can co-occur with INI-mediated increases in pragmatic competence (7).

In another interview during month three of INI, the researcher asked EJ about what he meant by his comment earlier that day that his wife and daughter were “independent observers” of him. He replied by using a joke (starting with a smile) which aimed to deflect criticism of his short-term memory loss and achieve a sympathetic (perlocutionary) effect on the listener (i.e., the researcher who then laughed):

“I meant have I changed in my ability”. [Researcher: “What have you heard about your abilities?”]. EJ: “Well, that I am forgetful (smiles). And I don’t do what they say sometimes” (laughs). “Ah, ok” [researcher also laughs]. EJ: “Sometimes, I am not totally forgetful. It is just that I have my own agenda, you know. Like today, before I knew it, I was washing out all the coolers. We had a number of them between us and some borrowed even. It goes pretty smooth…”

Treatment mediated self-depreciatory humor can deter aggressiveness and assert autonomy. This humor aims to influence conversational partners to share one’s view of a topic and/or to change a situation in which caregivers are intruding upon one’s privacy or autonomy (6, 14). EJ also employed sarcasm that day, noting: “My wife complains to me that I didn’t do something … *And …* it is always my fault” (8/9/21). Here EJ is showing self-awareness of his own limitations and appears to be using humor to protect himself from negative experiences.

### 4.5 Month 3: less irritable, complex cognitive load

At week seven (8/30/21), EJ said, when asked how he was doing: “Fine, slept well … I feel good”. When asked about the status of his memory, EJ replied that he had attended a party for his daughter’s housewarming which was “very nice, excellent turn-out. I find myself talking to all the people at the party. I try to remember names—and I cannot immediately, but then it will pop-out eventually— and then, of course, I try to use their name when I see them and they know me too by EJ, you know (smiles). So, it worked out though, the food was good, it was a good day. But, so, generally, I feel good”.

When asked if the patient still administered his treatment himself, EJ provided a detailed description of his process:

“I did my intra-nasal treatment today. I take my time; I double-check that I do the injector before I started the atomizer. I try to lock in a 9am-9pm hour cycle. I carry the box with me into the bedroom. I did some treatments … I put the insulin into the nose piece and breath it in until 99% of the liquid is gone. I am following the instructions on what to do. I feel refreshed after the treatment … Nothing feels bad. I put the (empty) needles into the vial; it goes smoothly.”.

In this interview with wife and daughter, the researcher asked: “What have you noticed about EJ? Is he the same, better or worse?”. EJ’s wife replied: “He is not as grouchy. He used to be grouchy and mean and get frustrated all the time and take it out on me—so that is better.” She and their daughter noted that EJ tends not to ask for help, perhaps to assert his autonomy, when looking for things. His daughter noted that, despite exhibiting frustration when he is unable to find an item, EJ doesn’t yell. However, she complained that his memory and orientation had not improved and had declined. She also reported that EJ lacked social participation.

### 4.6 Month 4: increased pragmatic competence (irony, jokes, less conversation withdrawal), yet continued sequence and step omission errors

At week 13 (10/26/21), the researcher re-interviewed EJ and his wife. EJ began the interview with: “I am feeling good.” He was also aware of his own anxiety about administering the treatment. EJ remembered that he had returned from a 4-week cruise to Greece a few months earlier. “That was kind of tiring … The worst part is the travel. The four connections we had then coming back home. It was not a big rush [while you are on the cruise]. You get up in the morning and go have lunch. It is easy to get fed. But at any rate, now I am back to a more normal week. I will play some golf next week.”. EJ was still driving and partially recounted how he acquired a new license around two months prior.

“I feel pretty good and I try to read stuff, you know, stay current. It is not like I sit there, staring at the wall [laughter]. I am busy.” EJ also talked about his retirement, commenting that he “was not bad off.” He also recounted to the researcher that a couple of friends had “passed over” [died] including his nephew; a friend he worked with and they were both younger than him. He noted: “That is the catch, though so I guess I am doing pretty good for getting as far as I have.”.

Wray (16) notes that it is often challenging for caregivers when an MCI or AD patient’s behavior no longer fulfills the (previous) expectations of a caregiver, spouse, child, or family member. To keep communication non-aggressive, caregivers must continually access empathy. Caregivers must interpret aggressive, withdrawn or otherwise confusing behavior from patients as the result of reduced cognitive capacity, memory loss and distress. Many are not able to access this empathy and instead “construe ‘normal’ without any account of the abnormal pressures that fuel extreme responses” (16:116–17). Communicative conflict ranges on a continuum from internal unease to external aggression.

EJ and his wife used sarcasm as a pragmatic strategy to deal with stress that arises when he was unable to complete basic household tasks (10/26/21). EJ’s wife entered the room and sat down. The researcher asked EJ: “Do you have any challenges still”? EJ’s wife interjected, sarcastically: “It took him four hours to make pancakes this morning.” This sarcastic utterance expresses resistance to EJ’s inability to meet her expectations of his cognition. She expected that EJ *should* be able to make pancakes as efficiently as before MCI diagnosis. As Wray (16:129) notes, caregivers can resist admitting the [new] level of the problem especially when loved ones now need aspects of parental care (36) and often suffer from endemic stress themselves.

In response, EJ employed self-depreciatory humor as an illocutionary strategy. Self-depreciatory humor can have a series of functions including deterring aggressiveness and achieving appreciation and sympathy. This humor can also function defensively to protect one from negative experiences (37). EJ replied: “It took me a couple of hours” [to make the pancakes], then pointed to his wife and said: “She has her own opinions.” EJ’s wife then remarked defensively: “He is looking at me.” At this point, EJ turned to researcher and said: “She harps at me all the time. Can you fix that”? This utterance induced surprised laughter from the researcher (a successful perlocutionary effect) to which the researcher replied: “That is not an insulin problem.” EJ’s wife then retorted: “Wait until she gets to [interview] me and I will tell her”. EJ again employed sarcasm as a pragmatic response: “I am sure she cares so much.”.

The use of sarcasm and irony require high cognitive loads and both pragmatic processes represent indirect performatives of a single utterance by way of performing another (38). INI improved the perlocutionary capacities of a patient with moderate AD who expressed humor to induce laughter (the intended perlocutionary effect on others) when defending intrusions on his autonomy by caregivers (5). Similarly, EJ employed sarcasm to reassert autonomy and control over the situation by use of pragmatic competence.

### 4.7 Months 5-7: falling back down; underdosing and concomitantly increased irritability, decreased sociability and short-term memory-related sequential tasking

Unfortunately, by late October, EJ began under-dosing his INI (see Supplementary Info). Consistent with findings on INI patients two months after treatment cessation (1), EJ’s caregivers subsequently reported increases in memory loss, social withdrawal and irritability by late December 2021. EJ’s wife reported that her husband had returned to being frequently “upset” and “hard to live with”. Regarding social withdrawal, she said: “When we were on [a cruise], EJ would at least talk to people at meals. A week ago, we went out to dinner with a couple and everyone was talking and he was just eating, sitting like a zombie. I thought: ‘What is hell is he doing here?”.

EJ showed increased short-term memory loss during the period of underdosage. In late December when the researcher asked him: “How are you doing?” EJ gave a long pause and then reported he felt “down” and was suffering memory issues. “I am a little bit senile; I don’t remember things” (12/29/21). His wife complained that her husband: “Didn’t even remember how to put the [atomizer] piece to his nose” and that he argued with her about the treatment process (“Just want to let you know how much he has deteriorated”) (12/30/21). When asked by the researcher: “Are you feeling any better”? EJ responded: “I think so. I am still able to argue with my wife. I am not suffering in any way, although I am forgetful.” His wife reported that her husband was now only able to complete tasks requiring one step (1/6/22).

### 4.8 Months 8–9: (re-)rise in procedural memory

Full dosage was restored on 12/30/21 (Supplementary Info). After five weeks, EJ displayed improved pragmatic capacities, self-awareness and empathy for others. After being notified of the impending arrival of a new atomizer, EJ responded to a group text with the researcher, his daughter and wife:

“Good to hear we have some progress on the new machine. I feel comfortable with the consistency of the current machine so hope the new machine will be even better? Thanks for your help.”

This response further displays self-awareness expressed as anxiety about potential future treatment issues (“I hope the new machine will be even better?”). EJ’s verbal response also incorporates illocutionary response (“Thank you for your help”), showing social and linguistic engagement.

By 2/2/22, EJ reported that he: “Felt good, physically strong … a little forgetful—sometimes I forget something in the evening that I might remember in the morning … When things get busy it is harder.” Nevertheless, the patient had begun, in the previous two weeks, to enter data on his rental property taxes into TurboTax. EJ had not been able to complete this task for over a year. Now, however, he noted: “You just follow the program, answer the questions, put income in there … It is a bit of a nightmare with the receipts and confusion but if you enter it right, it automatically puts it in right.” He thought he was doing this task “pretty good”. His wife concurred that he had successfully completed his taxes.

EJ reported that, earlier on the day of the interview, he had driven to Walmart, shopped at ALDI with a grocery list his wife had given him, and collected his mail. When asked about his social life, EJ noted that he watches TV, shops for groceries, “does not get out much now in winter with the snow” but feels “good”.

EJ self-administered his INI for two months, suggesting greater cognitive load that allowed him to complete the associated complex sequential series of steps. On February 8, 2022 (treatment month nine), EJ described details of this process to the researcher: “I keep checking the insulin level”, “I count how many breaths I intake”, and said he kept the atomizer on until all the insulin was inhaled. He also described the precise, accurate atomizer cleaning practices he was conducting daily^7^. EJ was again able to conduct detailed serial tasking.

Short-term memory tasks involving remembering novel information across brief intervals are difficult for patients with MTL damage (39). In February, EJ reported continued experience of “dead zones” in his memory relating to novel information. For example, when asked about communication with his wife, EJ explained: “I am pretty much there [but] usually when I am on the phone or giving out information, I get a dead zone, forgetting what I was talking about. It is easy to have a dead zone”.

Another example of a “dead zone” occurred when EJ called the researcher two days after the previous interview (February 10, 2022). When she answered it, no one was there. She called EJ back within several minutes and he asked: “Did I just call you?” When the researcher said “Yes”, EJ then asked for confirmation: “So I called you and hung up?” EJ then discussed his anxiety about ensuring that his new atomizer would arrive. Once reassured, he confirmed that he “felt good” and “should probably get back to TurboTax to get some work done.” In a text sent February 10, EJ remembered that he needed to acquire medical records requested by the researcher two days prior. Furthermore, EJ followed multiple politeness conventions in this conversation, asking the researcher: “Do you have any further questions?” and then, a few minutes later when the doorbell rang unexpectedly; “I better let you go, I gotta answer the door. Thank you.”

EJ’s use of social politeness (“Thank you”, “Do you have any further questions?”) achieved the perlocutionary intent of expressing appreciation for the researcher’s effort. His ability to achieve intended effects of performative utterances suggests intact pragmatic competence at that moment (7). This ability is in direct contrast to pragmatics of untreated AD where communication misfires are frequent. As David et al. (40) discuss, untreated AD patients forget many elements of daily experiences and personal narrative and become unable to use humor and sarcasm. In contrast, EJ recalled previously forgotten elements of his daily personal narrative (“I have dead zones”, “Did I just call you”) and asked for help (“So I called you and hung up”)? thus avoiding potential breakdowns in communication exchanges^8^.

### 4.9 INI treatment effects: MRI, CSF and cognitive testing

EJ’s baseline MRI showed abnormal grey matter loss in the temporal lobes consistent with high IR (**Figure 3A**). After treatment, EJ’s neurologist reported that his second MRI profile (**Figure 3B**) did not fit with a diagnosis of AD but rather early MCI (Patient’s Notes, 3/11/22). Neurologist notes reveal “Ventricles and sulci are within normal limits” (**Figure 3B**). Thus, after nine months of INI, EJ did *not* experience significant reductions in grey matter and GMV temporal lobe ventricles (relative to baseline, **Figure 3B**)^9^.

Evidence suggests that INI increases regional cerebral blood flow in the putamen, insular cortex and hippocampus (41, 42). Intra-nasal insulin can clear beta-amyloid (Abeta42) in the brain, which is hypothesized to enhance central nervous system signaling, thereby improving pragmatic functioning and selective attention (43:6). After nine months INI, EJ’s cerebrospinal fluid biomarkers were Abeta42 (pg/ml), 485.3 (Borderline Abnormal), Total tau (pg/ml), 727.1 (Pretty to Quite Elevated) and Ptau181 (pg/ml), 87.3 (Pretty to Quite Elevated) (Patient Test Results 3/11/22). According to neurologist (#2), EJ’s INI lowered his Abeta42 (pg/ml) to “Borderline Abnormal”, evidence of beta-amyloid reduction.

### 4.10 Cognitive diagnostic tests

**Figure 4:**
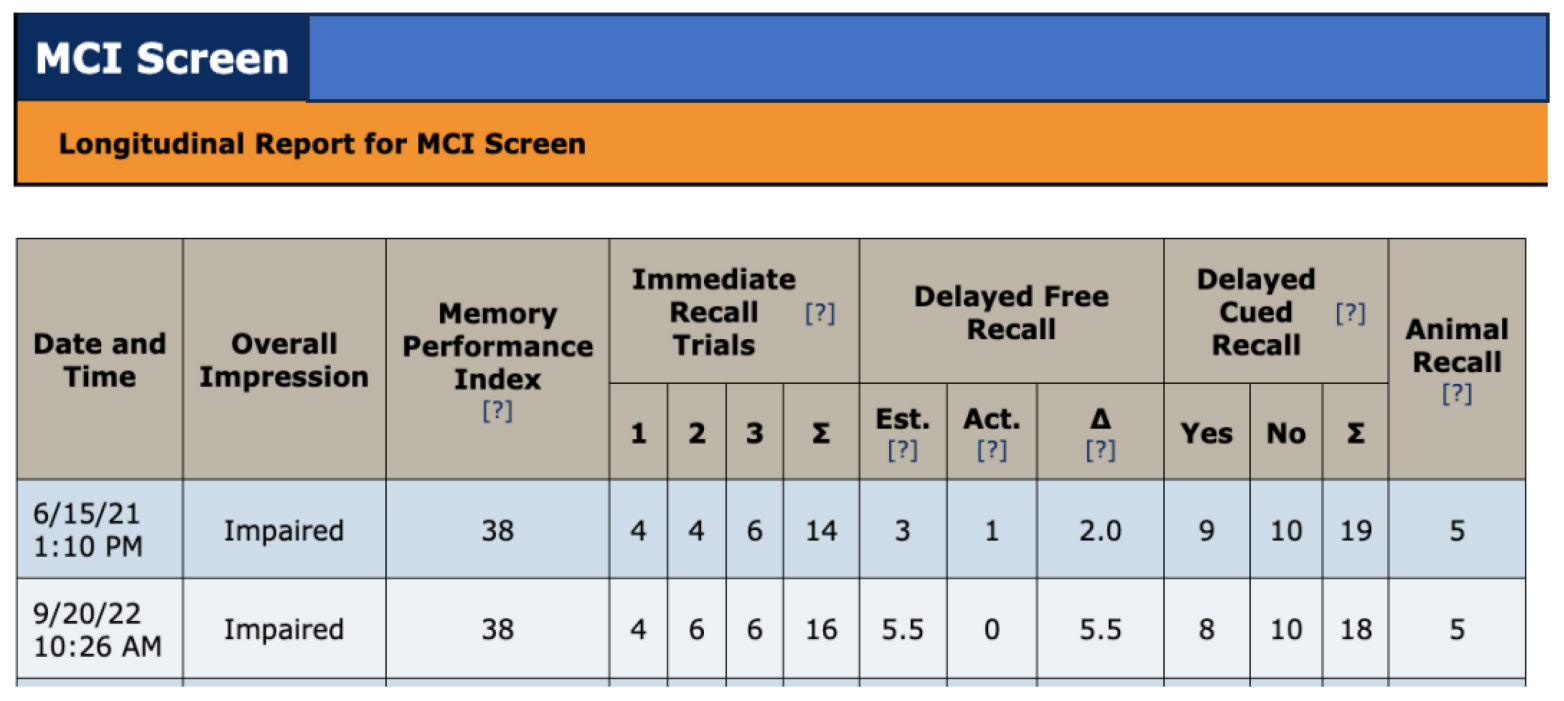
MCI screen administered at treatment baseline (6/15/21) and after 15 months treatment (9/20/22).

**Figure 5:**
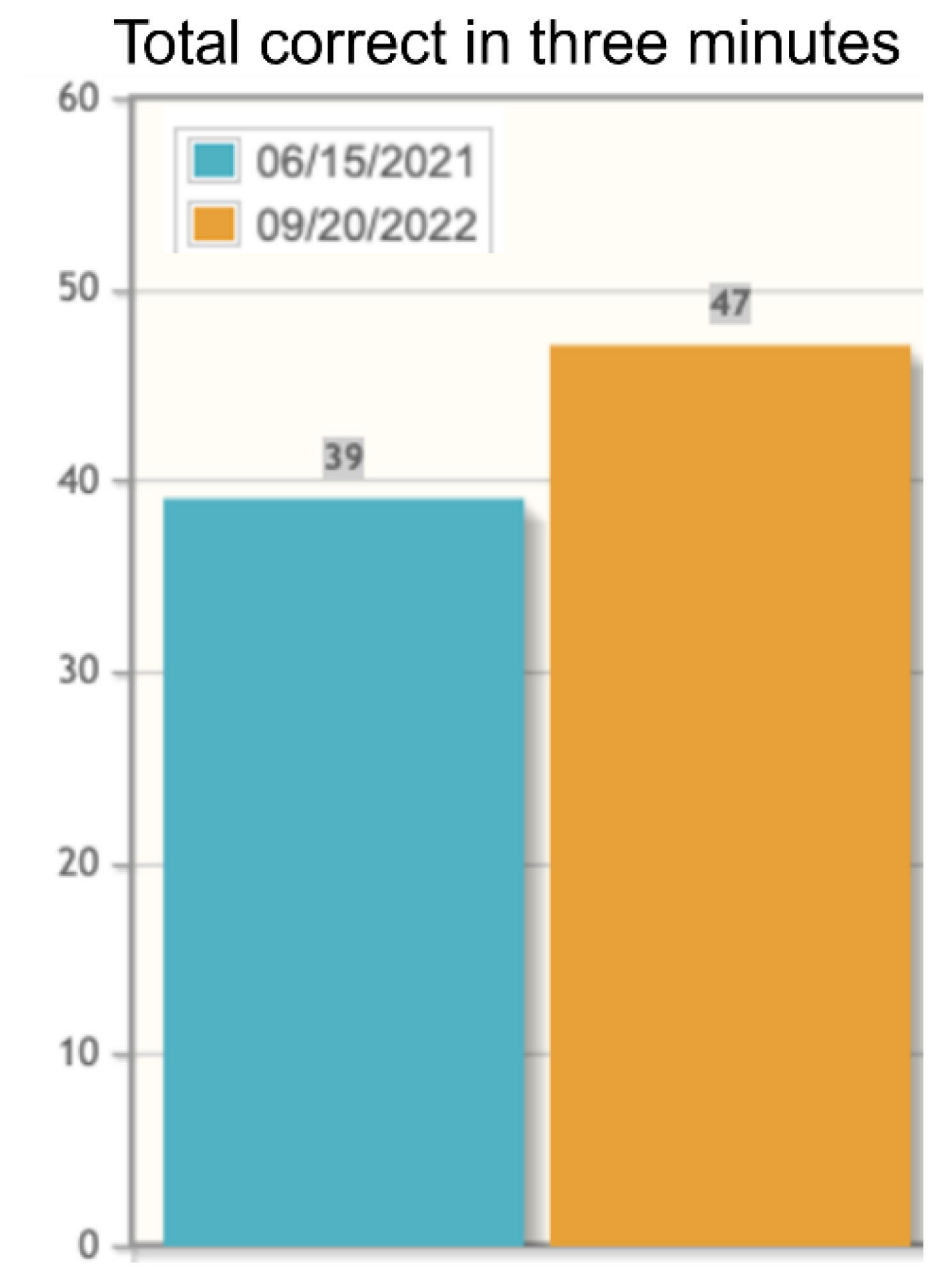
F-A-S Test of phonemic verbal fluency administered at baseline (6/15/2021, blue bars) and at month 15 of INI treatment (9/20/22, orange bars).

MCI screen (**Figure 4**) and F-A-S test of phonemic verbal fluency (**Figure 5**) for baseline and after 15 months of treatment. In the MCI screen (**Figure 4**), patient scores improved in immediate recall trials and in estimated delayed free recall. Scores remain the same as at baseline 15 months earlier in immediate recall, delayed cued recall and animal recall. The patient dropped 1 point in delayed free recall accuracy, delayed cued recall and in total delayed cued recall. FAS results show patient improvement in this phonemic verbal fluency test (**Figure 5**). These results are consistent with the INI clinical trials demonstrating cognitive improvements under INI treatment (3:**Figure 4**, 18 months, n=49).

## 5 Discussion

### 5.1 Contextualizing treatment-mediated improvements

INI improved pragmatic capacities, executive-functioning and procedural memory in an insulin resistant patient with Mild Cognitive Impairment (MCI). Improved pragmatic capacity included the achievement of positive (perlocutionary) effects, such as asking for help and clarification (“So I called you and hung up”)?, engagement in conversation, and determent of verbal aggression using sarcasm and humor. Treatment facilitated several pragmatic strategies for coping with disease related losses of memory and functionality (“I do a lot of backtracking”). Pragmatic improvements also included renewed ability of the patient to assert social, emotional, and medical needs to others (“I feel good,” “I am still able to argue with my wife”, “Thank you”, “I feel down”). INI thus reduced EJ’s social isolation as a speaker.

Pragmatic inference generation and executive-functioning are similar processes given that “increasing degrees of impairment in the executive system correspond to greater and greater impairment of inferential reasoning (44).” EJ’s use of figures of speech (“It is easy to have dead zones”) is an example of inference generation in pragmatic competence. Usage of figures of speech involves abstraction and the computation of meaning, skills which require high cognitive input and executive-functioning (5, 7, 14, 45). Specifically, figures of speech involve more efficient cognitive function within the default mode network [DMN] control, in conjunction with the frontoparietal network (46). This network is an area where the perfusion of intra-nasal insulin occurs (18) and is a key site for pragmatics (47).

Typically, persons with dementia are not able to retrieve and produce specific topics from memory and/or maintain personal agency in complex conversational exchanges. Their use of figures of speech, metaphors, metathesis, and sarcasm also typically decline with advancing AD stage (45). Rare occurrences of sarcasm, irony or complex figures of speech occur in patients with moderate and severe AD (48). Nevertheless, dementia patients are often assumed to lack the pragmatic competence to understand such utterances and “will be confused or even hurt by the intent if others use it” (49). At INI month four, late-stage AD patient KG was able to express a metathesis to the handyman working at her house who was complaining of fatigue (“Are you tired or *re-tired?*”; 14). She also expressed a sarcastic utterance while watching a romantic movie (“He is giving her a real line”), and declared, while listening to a song about falling in love (“That is not going to happen to me”) (14). Similarly, a moderate AD patient at diagnosis AR was still able, at treatment year five and a half (several weeks before his death), to employ bodily-related metaphors that required abstraction from literal meaning (compute related predicates) (6, 50). He said: “I am tired. It seems like that is all I can say. What a pain in the ass that is” ^10^.

INI targets the right anterior insular (rAI) cortex via increased cerebral blood flow (42)^11^. The rAI is an area directly related to subjective interoceptive awareness (self-awareness of distinct emotions and bodily feelings) and, by extension, emotional depth and complexity (53:241). As EJ explained in one interview: “I do get told to do a lot of things, and sometimes I do not do them all.” His wife interjected: “He can’t do a two-step thing; it always has to be one step” (10/27/21). EJ agreed and said:

“But part of the problem is that I don’t see *why* I am asked to do certain things that somebody else could have done on their own.” At this point, the researcher inquired: “Are you telling me you are aware and that you are making a conscious choice?” EJ replied: “I don’t remember really, but [pause] yea, I think it is, it gets out—I don’t remember to do it honestly but *it’s partly I think because I don’t really want to do it* [emphasis added]*”*).

Here EJ expresses volition, a component of executive-functioning, as well as subjective self-awareness, an aspect of rAI functioning (54). Introspection requires internally and externally directed attention, which are aspects of cognition. Executive function and inference require simultaneous attention and the processing of multiple sources of information in parallel to ensure planning, judgement and task completion (55:30). Clinical trials with INI show improved procedural memory in MCI patients at treatment month six (3). EJ was able to self-administer INI for two months with supervision and successfully execute the TurboTax program^12^.

One preventative measure for preserving pragmatic and cognitive load functioning may be early, consistent and targeted INI for mild MCI patients. After nine months of INI, one patient’s daughter said about her mother:

“She is diagnosed with mild cognitive impairment so although she has trouble with short term memory and organization, she is still somewhat independent and can still follow most conversations. If you did not know there was something wrong and you spent lunch with her you might not realize anything going on. It’s short-term … hearing something and retaining. *It’s almost as if she were not paying attention when you were talking to her. I do believe however that the device is helping her stay at this place* (7:33)”.

In EJ’s case, early detection of IR and earlier INI may have lessened his initial MTL tissue loss and advancing MCI status.

Nevertheless, advances in pragmatic and executive-functioning are INI dosage dependent. Cessation of INI rapidly reduces such advances in MCI and AD. Four to six months after treatment ended in the Phase 2, INI clinical trial, INI patients declined significantly on the Delayed Story Recall and the DSRS [Dementia Severity Rating Scale] (1). Similarly, after two months of under-dosing, EJ’s anxiety and confusion increased, and he lost previous procedural memory (ex. becoming unable to self-administer INI). EJ expressed his own deterioration in cognitive and pragmatic functioning (“I am a little bit senile,” “I don’t remember things”). He also verbalized high anxiety and concern about the future of his treatment (“What is the correct dosage?”, “What is going to happen to me?”) (12/28/21).

### 5.2 Considerations when administrating INI

EJ’s findings align with results from large clinical trials on INI and suggest that treatment can slow neurodegenerative disease progression in MCI patients. Clearly, the effectiveness of INI to treat MCI is driven in part by the method of delivery of INI (atomizer type & reliability), the dosage (twice daily, 20IU or 40IU) and the type of insulin (regular-Novolin R, Humulin R or long acting insulin detemir)^13^. ViaNase devices delivering INI in AD and MCI modified functional connectivity within memory network, improved cortical blood flow, enhanced vasoreactivity, cognition and improve functionality (58). Optimally, the atomizer must deliver a precise dosage targeted into the olfactory epithelium, and maximize nose-to-brain transport (59). ViaNase devices (Device 1) were continually used for 18 months in a subset of 49 participants in the large-scale Randomized Clinical Trial to investigate the safety, efficacy, and feasibility of intranasal insulin for the treatment of Mild Cognitive Impairment and Alzheimer’s Disease, published by Craft et al. in 2020 [Study of Nasal Insulin to Fight Forgetfulness (SNIFF) trial (using 40 IU of regular insulin (Humulin-RU-100; Lilly)]. Device I participants had significantly improved ADAS-cog-12 performance at 12 months (−2.81 points; 95% CI, −6.09 to 0.45 points; *P* = .09), improved Activities of Daily Living Scale for MCI ADL-MCI scores at month 18 (4.85 points; 95% CI, 0.07–9.63 points; nominal *P* = .05 (mean score change, -4 INI treated, -9 Placebo) (3:Figure #4a, c).

Furthermore, this SNIFF Trial cohort (n=49, ViaNase, 40 IU Humulin-RU-100) also showed “strikingly different patterns of associations between changes in CSF immune/inflammatory/vascular markers and changes in cognition, brain volume, and amyloid and tau concentrations upon analysis of CSF markers” (increased CSF interferon-γ (p= 0.032) and eotaxin (p= 0.049), and reduced interleukin-6 (p= 0.048) over the 12 month trial compared to placebo) (60:1346). In addition, this cohort showed reduced progression of white matter hyperintensity volume (60), which is a pathology linked to vascular injury and inflammation by amyloid or other factors. As Kellar et al. (60:1346) conclude: INI treatment “altered the typical progression of markers of inflammation and immune function seen in AD, suggesting that INI may promote a compensatory immune response associated with therapeutic benefit.”.

## 6 Conclusion

A model that incorporates pragmatic aspects (pragmatic units both as utterance elements and as units incorporating cognitive and socio-interactive dimensions) can shed light on the relationship between pragmatic and executive-function skills and can enhance MCI/AD communicative participation under targeted INI. INI can increase a patient’s pragmatic capacity via improved illocutionary and perlocutionary functions while enhancing cognitive load through internally directed attention, introspection and greater external attention capacity. Awareness of the complex and costly process of FDA drug-device approval are public health interventions needed to extend INI to a wider population (61). Preventing early MTL brain volume loss in IR and mild MCI patients remains critical to arrest dementia related declines in executive-functioning and pragmatic competence as well as reduce caregiver stress.

## Data availability statement

The raw data supporting the conclusions of this article will be made available by the authors, without undue reservation.

## Ethics statement

Written informed consent was obtained from the individual(s) for the publication of any potentially identifiable images or data included in this article.

## Author contributions

SS: Conceptualization, Data curation, Investigation, Methodology, Project administration, Resources, Supervision, Validation, Visualization, Writing – original draft, Writing – review & editing. GRG: Validation, Visualization, Writing – original draft, Writing – review & editing.

## Footnotes

1 This article also defines INI treatment as usage longer than 4 months. 3-4 months (short-term) was the standard time frame for Phase 2a FDA INI trial (1).

2 Schatz & Rivera (2016)’s model of the linguistic dimensions of the loss and return of perlocutionary and illocutionary functions of INI in MCI and AD patients understands language (pragmatics) as related to a wider social context, i.e., the patient with family, caregivers, in community settings. This model understands pragmatic units as utterance elements, which refers to the use of language to perform an action or to “do something”—perform an act of different sorts: thanks, apologize, defend oneself, express humor. An utterance only does something when uttered in the appropriate circumstances. There are three acts related to an utterance, first, the act of saying something meaningful (the locutionary act), the illocutionary act (the act performed in saying something) and the perlocutionary act or effect. For a longer theoretical discussion of the linguistic theory behind performatives and pragmatic function, see: Rexach & Schatz (7:325-329).

3 EJ’s glucose levels, ages 64-69: 115-10/3/06; 101-10/30/07; 108-11/04/08; 105-11/24/09; 111-12/7/10. Diagnosis Code: 790.29, R73.9, abnormal blood sugar, inconclusive without diagnosis, 2022 ICD-10-CM Diagnosis Code R73.9. Unfortunately, EJ’s medical records before 2006 are not available.

4 For the HbA1c test, “pre-diabetic” is a range of 5.7-6.4, diabetic is 6.4 and upwards. *EJ’s one normal HbA1c score after July 2018 corresponded to a 5lb weight loss from the prior test (10/20/20). EJ’s 2016-21 weight was: 186.1 (2/19/16), 179.8 (5/3/16), 181.1 (1/31/17), 183.8 (3/21/17), 182 (3/5/18), 179.5 (5/4/18), 176.9 (7/31/18), 175.3 (10/24/18), 170.0 (4/30/19), 173 (10/08/19), 171 (10/20/20), 167 (3/4/21), 164 (4/6/21). HbA1c tests were not conducted before 7/31/18, the period of higher weight (182-186lbs, 2016-2018).

5 EJ’s pre-diabetic HbA1c test state increased to a diabetic state (6.4 HbA1c test, 7/29/22). EJ’s glucose level was 201 mg/dL (7/29/22). Becerra et al. (29:828-829) note: INI “did not regulate appetite or peripheral energy expenditure and could not correct insulin resistance in participants with type 2 diabetes. The lack of INI effects on body weight, BMI, glucose and HbA1c is consistent as seen the whole cohort of the MemAID [T2DM] trial (25)…A dose-response curve of INI effects on body metabolism may be needed to determine if any effects could be seen at higher INI doses (2023: 832)”.

6 The FEDA test (subjectively experienced deficits in attention performance) delineates such categories as focused attention, vigilance, and divided attention-visual or auditory to evaluate attention improvement after clinical intervention (35).

7 On 2/8/22, EJ also described to the research how he had fixed a minor hose problem (“I re-inserted it into its slot”). He also suggested to the researcher that she could “tape” the interview when she said that she needed EJ to slow down his talking so she could take written notes to follow his verbal flow.

8 EJ’s wife also reported another decrease in EJ’s irritability, an increase in his social conversational abilities in social settings and a decrease in her care-giver stress levels after the increase in dosage (12/28/21-3/3/22). She reported EJ was now able to fully, properly engage in social conversation with family in a restaurant setting (3/3/22).

9 Doctor’s notes also report: “The brain is unremarkable structurally with no evidence of congenital or morphologic abnormalities. No acute parenchymal signal abnormality is identified. No evidence of edema. No evidence of mass lesion. No evidence of hemorrhage. No diffusion restriction or evidence of acute infarct. No extracerebral collection. Sellar and parasellar structures are unremarkable. Posterior fossa is unremarkable. Extracranial structures are unremarkable.” There are mild patchy FLAIR signal changes noted in the white matter, which are consistent with age-related microvascular ischemic changes. These are also within normal limits for the patient’s age (3/11/2022)”. Neurologist #2 placed patient on Namenda ER (20mg), DHA (1000mg), Percepta (2 capsules) and Metformin (500mg).

10 AR was also able to remain in his home with nurse administered INI for 5 years after AD diagnosis, finding and living with a new, elderly girlfriend (age 80-86). AR had been widowed a few years earlier. Even during the last year of his life in an assisted memory facility, he continued to eat by himself, interact with the mild AD cases in the nursing home, and was ambulatory with very minor assistance (51).

11 The insular cortex is connected to the DMN and acts as a “switching gate” between it, the salience network, and the executive-function network. Switching performed by the insular cortex allows sensory input to be evaluated by the desired network (52).

12 Similarly, a neurologist, upon posthumously examining “AR’s” CT scan after five years on INI, declared: “This man does not look like he had Alzheimer’s disease” (Dr. Melvin Gonzalez-Rivera, personal communication, 2019). The MTL, as a “hub”, includes the hippocampus and entorhinal regions.

13 In trials with MCI patients, insulin type (e.g., detemir insulin, ViaNase devices) and dosage can produce different treatment outcomes. INI can also have different effects at higher dosage levels (40 IU), even producing insulin-resistant metabolic effects and episodic memory decline in APOE - ϵ4 carriers (23:904). Interactions with gender and APOE ϵ4 carrier status with regular insulin at higher dosages (40 IU) (Novolin R, 1 dose a day, 4 months, ViaNase device) were also found. APOE - ϵ4 in women did not show cognitive improvement as compared to APOE - ϵ4 men, at 40 IU but these gender differences in APOE ϵ4 status did not have any effect at the lower 20 IU dosages (56:794). Conversely, plasma (Extracellular Vesticle Biomarker) studies of Craft et al. (1 data) participants show the engagement of the insulin cascade by intranasal insulin may be strongest in ϵ4 non-carriers who show the greatest insulin resistance at baseline at lower doses (20 IU, rather than 40 IU, Novolin R) (57:6).
