# Supplementary material for: Enhancing socio-communicative functions in an MCI patient with intra-nasal insulin: a case report
Source: Front Psychiatry. 2024 Jun 28;15:1326702. doi: 10.3389/fpsyt.2024.1326702 (PMC11239438; doi:10.3389/fpsyt.2024.1326702)
Supplement: Supplementary file 1 [file DataSheet_1.docx]

**Supplementary Information**

Loss of cognitive and affective functionality after treatment underdosing and/or cessation suggests lessened neuronal connectivity in key brain regions. Avoiding patient “brown outs” requires consistent patient education, device maintenance, and proper dosing. Device design can impact ease of use for patients and caregivers, which can affect dosing.

For the entire duration of this study, the patient used insulin atomizer devices of the same make and model with the same internal mechanism of insulin delivery. However, due to limited availability, the design of the device body differed between devices. Devices featuring the first design included an atomization chamber where each individual dose of liquid insulin was measured exactly and inserted by the user prior to delivery. The chamber had a clear cover so that when the device was in use, the patient and caregiver could see the level of liquid in the device diminish as the insulin delivered intranasally. Thus, the patient and caregivers could visually confirm after each treatment that the insulin had indeed left the device. In contrast, devices featuring the second design included an opaque upper housing unit where an entire bottle of liquid attaches. The housing unit was difficult to open after the bottle was attached. Also, each bottle contained enough insulin for approximately 8–9 days of treatment and the bottle did not have any measuring markings. There was therefore no way to denote the exact amount of liquid which should remain after each administered dosage. Thus, the level of liquid remaining in the bottle after each treatment did not reliably indicate that insulin had left the device as intended. If a device malfunction occurred which impacted the ability for insulin to leave the device, neither the patient nor the caregiver would have an immediate visual cue (i.e., that liquid insulin remained in the atomization chamber after treatment) to alert them of the issue. When EJ’s machine began to malfunction in late October, neither the patient nor his caregiver was able to detect that insulin was no longer delivered in the designated treatment amount until he noticed that he could feel no liquid at all entering his nose. His wife reports him saying “Nothing’s coming out [of the bottle]”. She did not alert the researcher of this malfunction until two months later (December 2021). As discussed in Section 4.1.8 of the main manuscript, when the researcher was informed, EJ’s device was immediately replaced with one containing the original design.

In sum, modifications of INI devices which permit caregivers to immediately confirm each dose administration may allow for early identification of device malfunctions. Prompt communication (between caregivers, family, researchers, manufacturer and other relevant parties) of such malfunctions is critical.
